# Get the News Out Loudly and Quickly: The Influence of the Media on Limiting Emerging Infectious Disease Outbreaks

**DOI:** 10.1371/journal.pone.0071692

**Published:** 2013-08-26

**Authors:** Anna Mummert, Howard Weiss

**Affiliations:** 1 Mathematics Department, Marshall University, Huntington, West Virginia, United States of America; 2 Mathematics Department, Georgia Institute of Technology, Atlanta, Georgia, United States of America; Public Health Ontario, Canada

## Abstract

During outbreaks of infectious diseases with high morbidity and mortality, individuals closely follow media reports of the outbreak. Many will attempt to minimize contacts with other individuals in order to protect themselves from infection and possibly death. This process is called social distancing. Social distancing strategies include restricting socializing and travel, and using barrier protections. We use modeling to show that for short-term outbreaks, social distancing can have a large influence on reducing outbreak morbidity and mortality. In particular, public health agencies working together with the media can significantly reduce the severity of an outbreak by providing timely accounts of new infections and deaths. Our models show that the most effective strategy to reduce infections is to provide this information as early as possible, though providing it well into the course of the outbreak can still have a significant effect. However, our models for long-term outbreaks indicate that reporting historic infection data can result in more infections than with no reporting at all. We examine three types of media influence and we illustrate the media influence with a simulated outbreak of a generic emerging infectious disease in a small city. Social distancing can never be complete; however, for a spectrum of outbreaks, we show that leaving isolation (stopping applying social distancing measures) for up to 4 hours each day has modest effect on the overall morbidity and mortality.

## Introduction

The goal of this paper is to provide a theoretical foundation for how the media and public health agencies together can significantly reduce the morbidity and mortality of outbreaks of infectious diseases. In response to media reports of outbreaks, many individuals take steps, including social distance, to protect themselves and their immediate families from severe infection and significant chance of death. Social distancing refers to individuals attempting to minimize or eliminate contacts with others (those outside of their immediate family), and can take many forms, including restricting or ending socializing and travel, and using barrier protections. For example, during the 2002–2003 severe acute respiratory syndrome (SARS) outbreak, social distancing behavior included restricting local and long-distance travel, using face masks, and using extra disinfectants [1]. Social distancing for sexually transmitted diseases typically includes using barrier protections [2]. Both face masks and hand hygiene have been shown to be effective social distancing measures for influenza [3].

We use the term media in a broad sense to include any news reports, ranging from public health agency announcements to social media sites such as Facebook or Twitter. Any of these reports may contain false information and rumors. For example, in March 2003, the Center for Disease Control and Prevention recommended that all non-essential travel to SARS infected areas be canceled (recommendation made March 17, 2003, reported by The New York Times March 18, 2003 [4]), while in Chinatown, New York, a false internet rumor spread that a local restauranteur had died from SARS [5]. Both reports led some people to cease travel to these areas, though only one report was correct.

Our models elucidate the effect of the media on an individual's decision to employ social distancing measures and the implications to an emerging disease outbreak. Our goal is not to construct predictive models but instead to lay a theoretical foundation to study the media's influence. We derive formulas for key epidemiological quantities that allow us to study their dependence on the intensity of the media's influence. We carefully examine and illustrate three different scenarios for the media influence through a simulation of an outbreak of a generic emerging infectious disease in a small city. Our models definitively show that public health agencies working together with the media can significantly decrease the severity of an outbreak by providing timely accounts of the numbers of new infections and deaths.

We begin with the classical and widely used 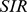 compartment model [6], [7] for the transmission of an infectious disease. To study the media influence and resulting social distancing, we add the following crucial assumption to the 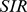 model:

### The rate at which individuals choose to employ social distancing measures is an increasing function of the number of current infections reported by the media

We implement the social distancing as a fourth compartment (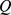) in the 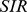 transmission model. Social distancing moves an individual from the susceptible class directly to the social distancing class and they play no role in the disease transmission. However, it is clearly unrealistic for an individual to remain completely isolated for weeks or months: individuals must venture out into the public to buy food and some will require medial care. Our long-lived models explicitly allow individuals to return from the social distancing class to the susceptible class. Our simulations show for a short-lived outbreak, that allowing individuals to return from isolation (stop applying social distancing measures) has little effect on the attack rate and maximal daily prevalence when individuals are allowed to leave for up to 4 hours each day. Therefore we focus on a short-lived outbreak with strict social distance (no return from social distancing) where we can do analytics. This seems to be the first study to address the question of partial social distancing.

There are other models incorporating the influence of human behavior on epidemics of infectious diseases. A review is provided by Funk, et. al [8], but there is a paucity of data. Several authors [9]–[16] modify the transmission rate in response to some gained information. Our models incorporate a different paradigm and does not change the transmission rate; they instead reduce the number of susceptible individuals. Other authors [10], [17], [18] consider vaccination strategies, which can be considered as a form of social distancing. Our models can be viewed as vaccination models where the number vaccinated is proportional to the number of infections and death reported by the media. In addition, they also accounts for any other social distancing measures.

Some governments are not always initially forthcoming with timely and accurate news of infectious disease outbreaks. We consider two types of time delays in the media reporting of an outbreak. If the media reports current infection data, then even starting to report well into the course of an outbreak can significantly reduce the severity of the outbreak. However, the long-term models indicate that reporting historic infection data can result in more infections than with no reporting at all (Figure S2).

In the body of the paper, we provide a non-technical description of the models and present the main results for short-term outbreaks. The precise statements and mathematical analysis are all delegated to Text S1. We develop a parallel foundation for long-term outbreaks (Figure S1), which is also in the Supporting Information. Our goal for this organization is to make the results of this paper accessible to non-modelers, while also developing and presenting the mathematical details.

## Results: A Short-Lived Outbreak

In this section, we study short-lived outbreaks and we delegate the discussion of long-lived outbreaks to Text S1.

A key threshold characteristic of an infectious disease outbreak is the basic reproduction number 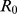, which measures the number of new infections caused by each infected individual at the beginning of the outbreak. Assuming initially that the fraction 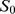 of the population is susceptible, we verify that the basic reproduction number in all models is
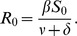



Our mathematical analysis shows that the infection dynamics are qualitatively similar for all three of the media influence functions. More precisely, if 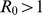, then the number of infections initially starts to increase. The number reaches a maximum and then decreases to 0. The classical 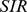 model exhibits the same behavior. Though the behaviors of the models are qualitatively similar, quantitatively they are different.

In the following, 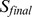 denotes the final (limiting) fraction of the susceptible population.

### 

#### Media Influence Function 1


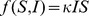


We derive the following formulas for the key epidemiological quantities.

#### Lemma 1

*• The maximum daily prevalence (fraction) is*



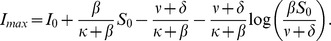



*• The attack rate (the total fraction of individuals who become infected) is*



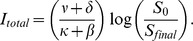



#### Consequences for the outbreak

We compute the dependence of these quantities on the media influence intensity, 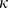. The derivatives of 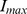 and 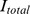 are negative which implies that as the media influence intensity increases these will decrease.

#### Simulations for an outbreak in a small city

**Figure 1:**
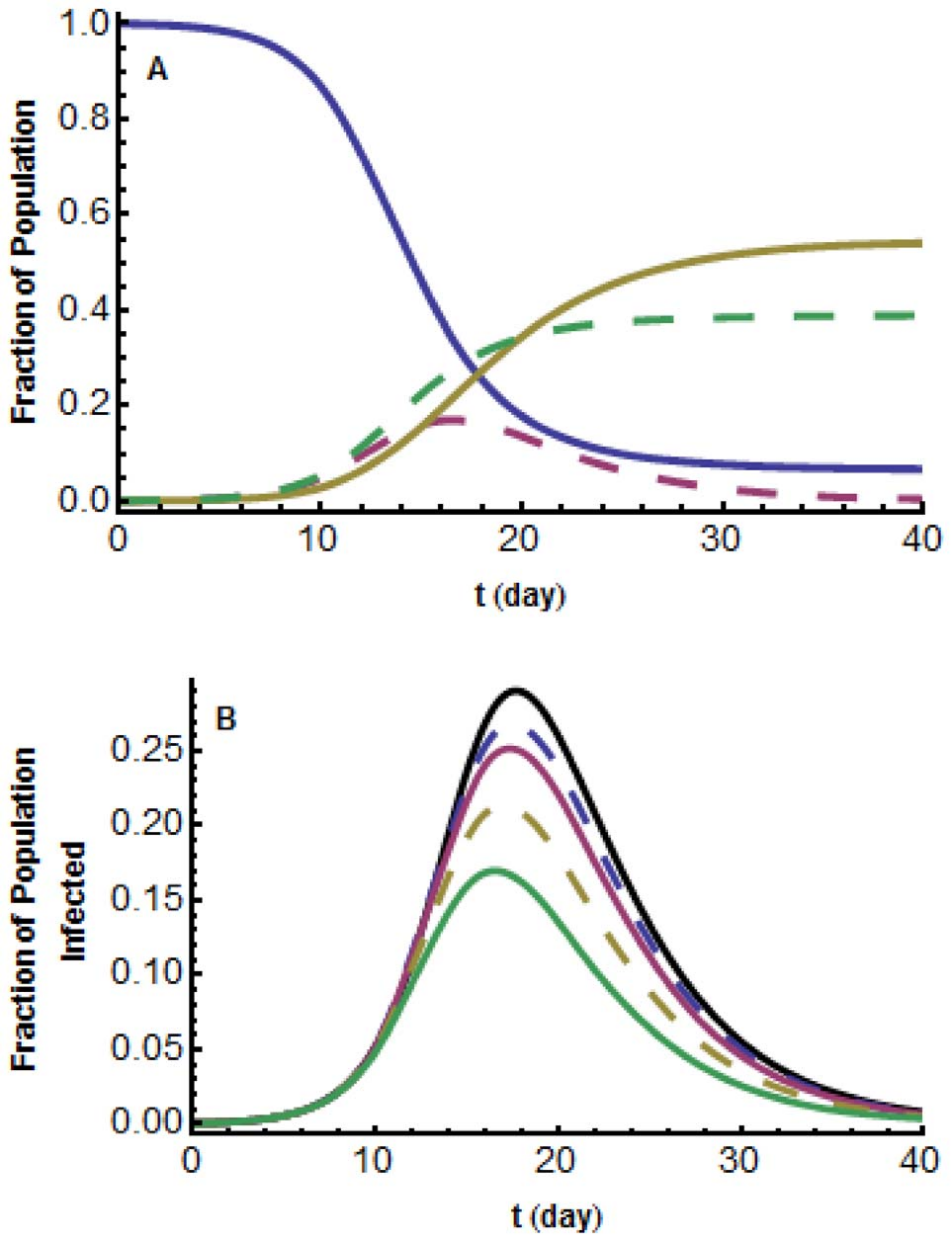
Short-lived model, 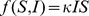. (a) Graphs of 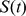, 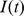, 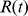, 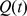; 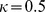; 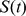 solid blue; 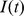 dashed red; 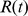 solid yellow; 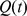 dashed green. (b) Graphs of 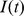 for five different intensities of media influence. From the bottom up: 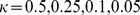. These are compared with the classical 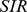.

Figure 1(a) shows the fractions of individuals in each population compartment during the disease outbreak. Figure 1(b) illustrates the effect of increasing the media influence intensity, that is increasing 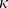. The resulting 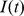 curves are compared with 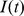 for the classical 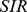 model. As can be seen, as the media influence intensity increases, the fraction who become infected decreases due to more and more individuals choosing to employ social distancing measures , and the maximum daily prevalence, 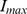, and the time of 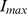 both decrease, which agrees with our mathematical predictions.

#### Media Influence Function 2


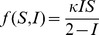


We derive the following formula for the maximum daily disease prevalence.

#### Lemma 2

*• The maximum daily disease prevalence is*



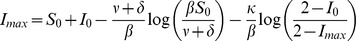



Due to the more complex form of the media influence function it is not possible to derive a formula for 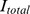.

#### Consequences for the outbreak

We compute the dependence of the maximum fraction of infected individuals on the media influence intensity, 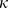. The derivative of 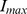 is negative which implies that as the media influence intensity increases the maximum fraction will decrease. The dependence of 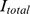 on the media influence intensity can be determined numerically for the parameters corresponding to the 1918 H1N1 pandemic influenza in the United States (Table 1). For the values in Table 1, we verify numerically that 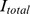 is a decreasing function of 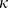. Without a formula, any other set of model parameters would need to be checked individually.

**Table 1:** Parameter descriptions, units, and the values used in model simulations.

Parameter	Description	Units	Value	Source
*S*(0)	initial fraction		0.9994	assumption
	of susceptible			
*I*(0)	initial fraction		0.0006	assumption
	of infected			
*R*(0)	initial fraction		0	assumption
	of recovered			
*Q*(0)	initial fraction		0	assumption
	of socially distanced			
*β*	transmission rate	day^−1^	0.7	assumption, [19]
*ν*	recovery rate	day^−1^	1/5	assumption, [19]
*δ*	disease death rate	day^−1^	0.04	assumption, [19]
*N*	total population	people	50,000	assumption
*κ*	media influence intensity	day^−1^	variable	assumption

#### Simulations for an outbreak in a small city

**Figure 2:**
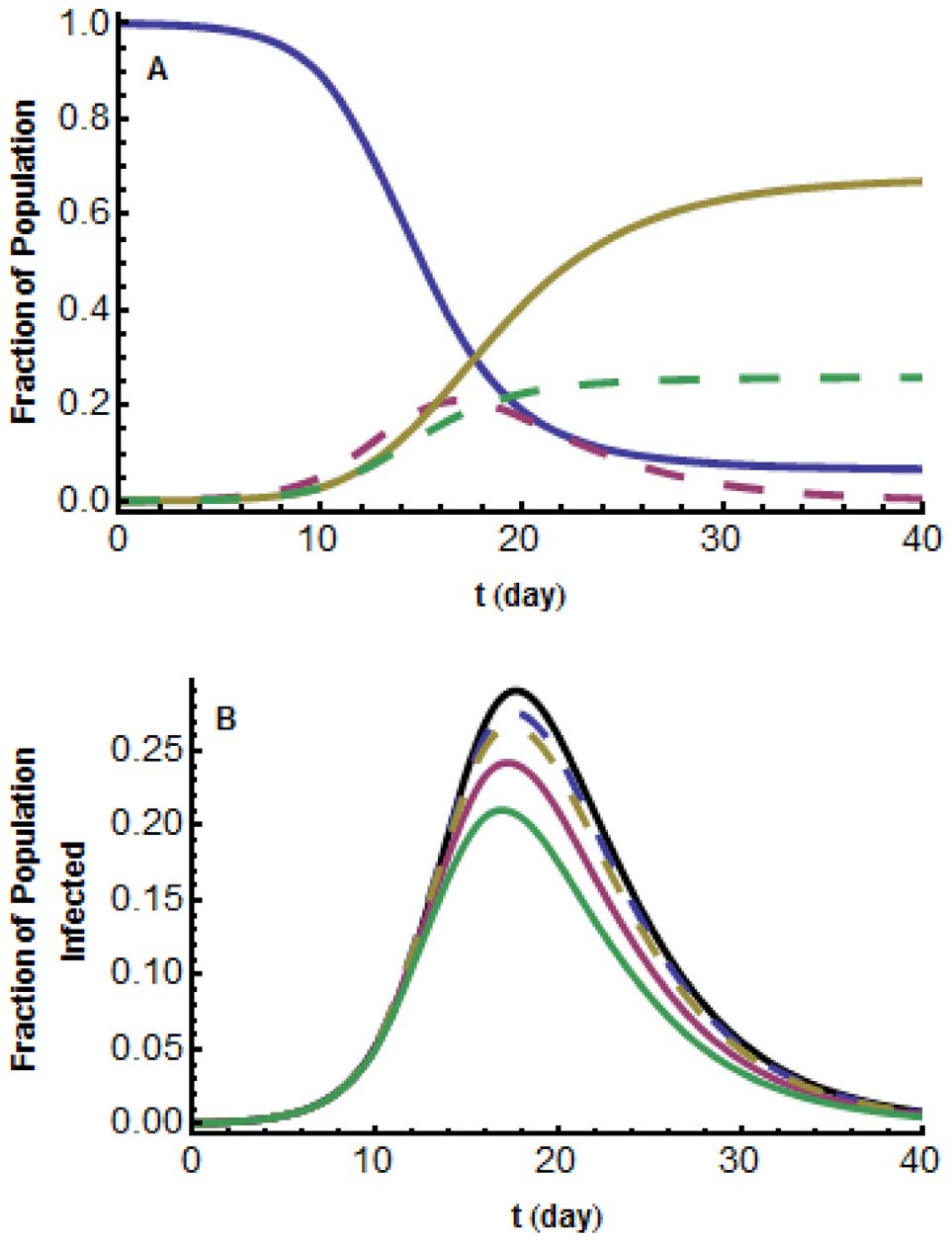
Short-lived model, 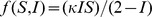. (a) Graphs of 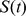, 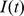, 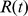, 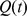; 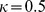; 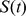 solid blue; 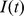 dashed red; 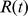 solid yellow; 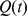 dashed green. (b) Graphs of 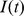 for five different intensities of media influence. From the bottom up: 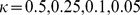. These are compared with the classical 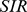 model with no media influence, 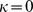, (top black curve).

Figure 2(a) shows the fractions of individuals in each population compartment during the disease outbreak. Figure 2(b) illustrates the effect of increasing the media influence intensity, that is increasing 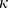. The resulting 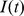 curves are compared with 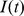 for the classical 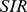 model. As can be seen, as the media influence intensity increases, the fraction who become infected decreases due to more and more individuals choosing to employ social distancing measures, and the maximum daily prevalence, 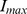, and the time of 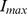 both decrease, which agrees with our mathematical predictions.

#### Media Influence Function 3



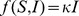



We derive the following formulas for the key epidemiological quantities.

#### Lemma 3

*• The maximum daily disease prevalence is*



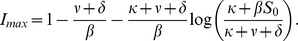



• *When 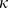 is less than the threshold 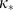, the attack rate is*



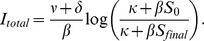



Due to the simplicity of the model, a pathology will occur if the media influence intensity is too high. For sufficiently strong media influence, that is for all 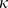 larger than some threshold 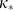, so many people employ social distancing measures that the number of susceptible individuals becomes zero in finite time. Since perfect social distancing will not happen even for the most frightening emerging diseases, this case is irrelevant for the purposes of the model.

#### Consequences for the outbreak

We compute the dependence of these quantities on the media influence intensity, 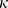. The derivatives of 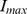 and 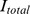 are negative which implies that as the media influence intensity increases these will decrease.

#### Simulations for an outbreak in a small city

**Figure 3:**
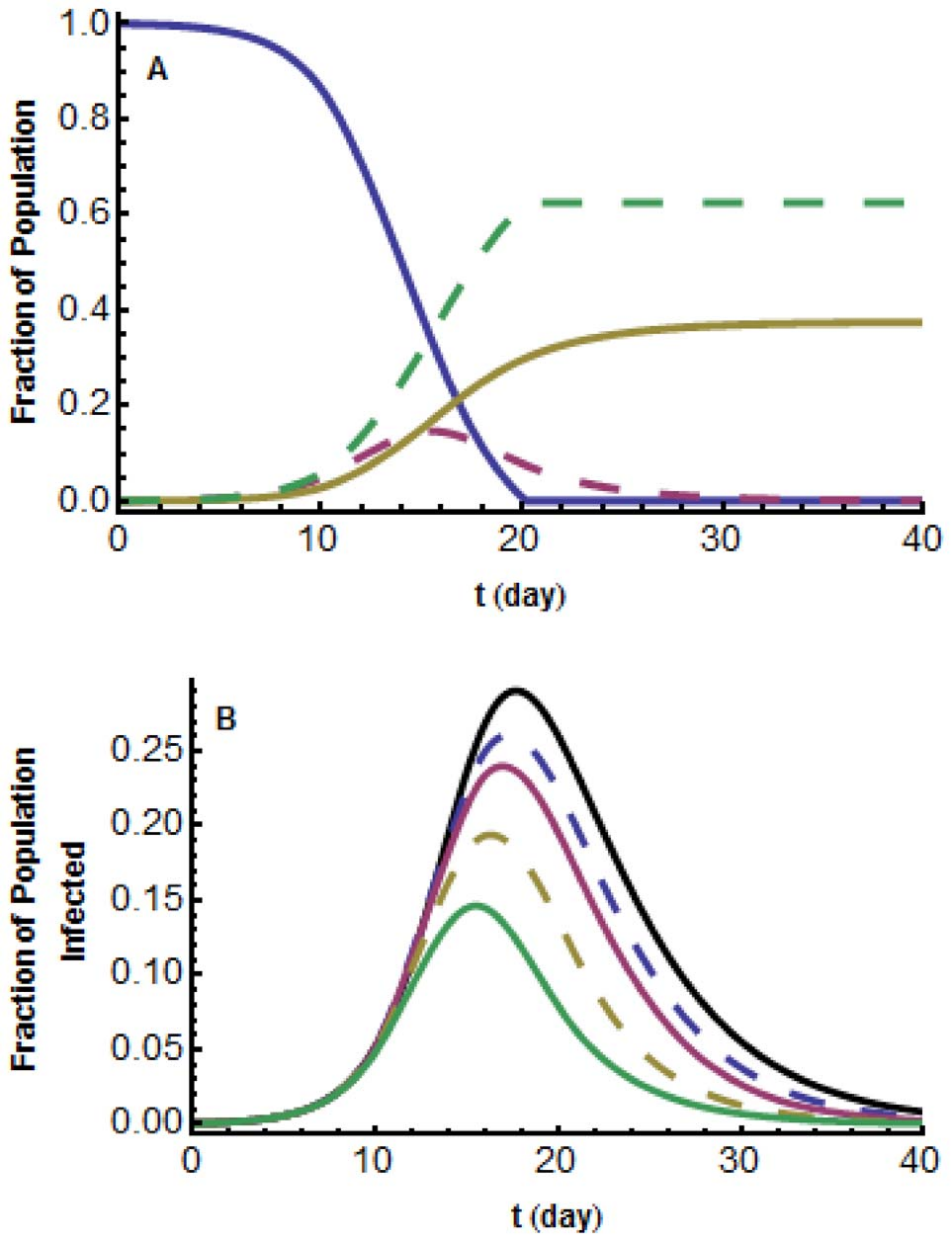
Short-lived model, 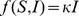. (a) Graphs of 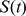, 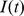, 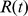, 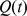; 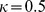; 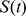 solid blue; 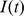 dashed red; 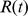 solid yellow; 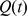 dashed green. (b) Graphs of 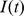 for five different intensities of media influence. From the bottom up: 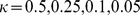. These are compared with the classical 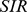 model with no media influence, 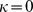, (top black curve).

Figure 3(a) shows the fractions of individuals in each population compartment during the disease outbreak. Figure 3(b) illustrates the effect of increasing the media influence intensity, that is increasing 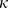. Several intensities of media influence, both above and below the threshold value 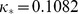, are shown. The resulting 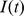 curves are compared with 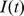 for the classical 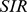 model. As can be seen, as the media influence intensity increases, the fraction who become infected decreases due to more and more individuals choosing to employ social distancing measures, and the maximum daily prevalence, 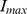, and the time of 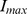 both decrease as 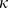 increases, which agrees with our mathematical predictions.

### Short-lived outbreak with non-strict social distancing

**Figure 4:**
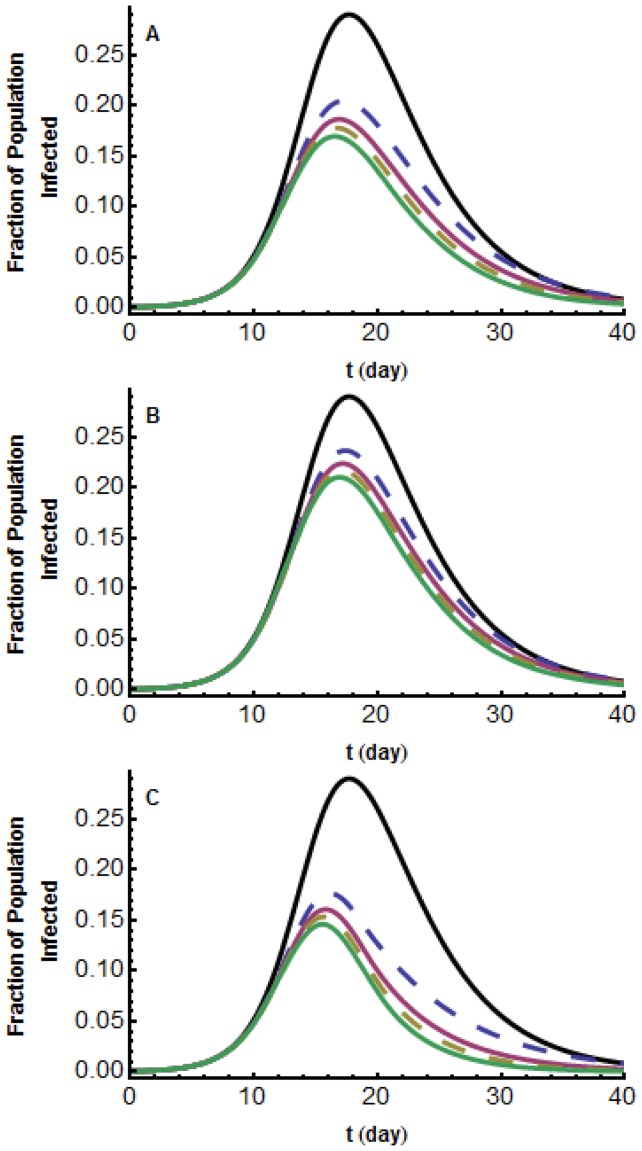
Short-lived model; 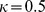; graphs of 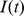 for different lengths of time out of isolation. Graphs of 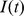 with media influence (a) 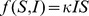 (b) 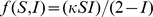 (c) 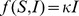. From the bottom up: time outside 0, 2, 4, 8 hours per day. These are compared with the classical 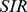 model with no media influence (top black curve).

Up to now, we have assumed that social distancing is strict in the sense that individuals and their families never leave their isolation. Since this is not realistic, we use the model to study the case where individuals are allowed to leave their isolation for some number of hours per day to obtain food, supplies, and medical care. As can be seen (Figure 4, Tables S1 and S2), for a large range of parameters, 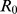 from 1.5 to 3.5 and 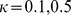, allowing individuals to return from isolation for 4 hours per day has modest effect on the attack rate or maximal daily prevalence. According to our simulations, in the worst case there is a 12% increase in morbidity.

For the extended model that includes a return from isolation, formulas for the attack rates cannot be determined. The attack rates must be computed numerically using the formula 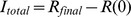, where 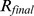 denotes the final (limiting) fraction of the recovered population.

### The effects of reporting delays

We extend our previous models to consider delays in media reporting. Including any such delay, creates a model that is infinite dimensional and seems analytically intractable. We are forced to rely on simulations to make any conclusions.

**Figure 5:**
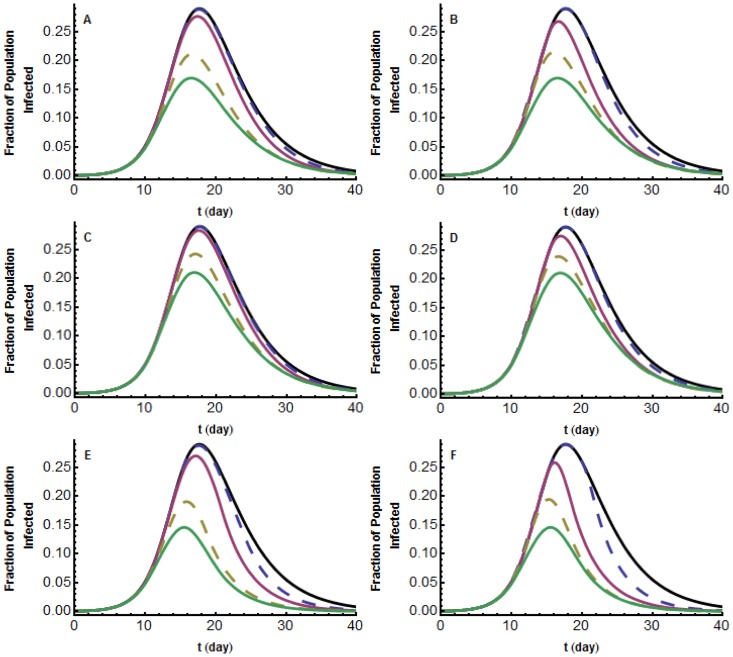
Short-lived model; 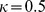; graphs of 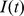 for different lengths and types of delays. Graphs of 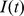 with media influence (a,b) 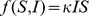 (c,d) 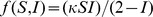 (e,f) 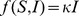. (a,c,e) Delay Type 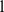. From the bottom up: delay 0, 2, 7, 12 days. (b,d,f) Delay Type 2. From the bottom up: delay 0, 12, 15, 20 days. These are compared with the classical 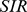 model with no media influence (top black curve).

For all three types of media influence, we simulate the effect of the two types of delays in media reporting, using the model parameters for the 1918 H1N1 pandemic influenza in the United States (Table 1) and 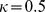 (Figure 5). In every case, as the media holds back information for longer periods of time, the attack rate increases and the maximum fraction of infected individuals at any one time, 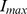, increases, as well as the corresponding time 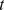 of 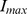. Furthermore, the fraction who choose to employ social distancing measures decreases.

## Discussion

We have developed a theoretical foundation for studying how the media and public health agencies together can influence the morbidity and mortality of outbreaks of infectious diseases. We begin with the standard compartment model for transmission and include social distancing of immediate families according to three different types of media influence. Our mathematical analyses show qualitatively that the infection dynamics are the same for all three media influence types. Both our theoretical analysis and our simulations for a generic emerging infectious disease outbreak in a small city show that increasing the media influence intensity will reduce the severity of the outbreak. Since this conclusion is robust for a large range of media influence types this provides confidence in the models' conclusions.

We use our models to study the effect of delays in media reporting and we consider two different types of delays. Simulations show that for all three models the infection dynamics are qualitatively similar for each type of delay. We conclude from our models that media influence can play a significant role in reducing disease prevalence. It will be most effective if started early in the outbreak, but still will reduce prevalence if started late, even if the peak of infection has already been reached. Our models show that if reporting starts late, it is more advantageous to report current data than historical data.

When the media influence intensity 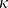 is small in any of the models, the media has little influence on the decision of immediate families to employ social distancing measures during the course of an outbreak, while when the media influence intensity is large the media has a greater influence on the decision of immediate families to employ social distancing measures. The smaller the fraction of the population that does employ social distancing measures, the smaller the media influence intensity 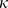. For a particular infectious disease outbreak, in locations where governments tightly control the media and decide to under-report the number of infectious and deaths, the media influence intensity will be small.

Although it is not easy to measure 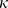, it is always true that maximal reporting will be the most effective in reducing the severity of the outbreak. Our models establish that purposely with-holding information on infections and deaths will lead to much unnecessary morbidity and mortality.

Strict social distancing is unrealistic because individuals or family members need to obtain food, supplies, and medial care. Therefore, we extend our model to allow for return from isolation for some number of hours each day. Our simulations of a generic emerging infectious disease show that allowing individuals to leave their isolation for up to 4 hours each day has at most a modest effect on the morbidity, for a wide range of parameter values, 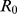 from 1.5 to 3.5 and 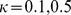. In all three models we observe that the excess morbidity increases as 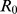 increases and as the media influence intensity 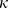 increases. Thus, the outcomes of our models with strict social distancing provide a good approximation to the more realistic case of incomplete isolation. We know of no other study of non-strict social distancing.

We stress that we did not attempt to construct a predictive model, which likely does not exist. We followed Occum's razor and constructed a simple model which captures the desired phenomena and is highly amenable to mathematical analysis. In particular, the model does not include age structure or heterogeneous mixing, and all social distancing actions are combined into one class. We derive explicit formulas for the dependence of the attack rate and the maximum daily prevalence on the intensity of the media's influence. We do so for all parameter values, and thus obtain a quite general understanding of the media influence on the morbidity and mortality. We conclude that the best policy for limiting infectious disease outbreaks is to *get the news out loudly and quickly*!.

## Methods

Here we describe the model for short-lived outbreaks, the media influence types considered, the possible delays in media reporting, and the simulation techniques.

### Modeling a short-lived outbreak

**Figure 6:**
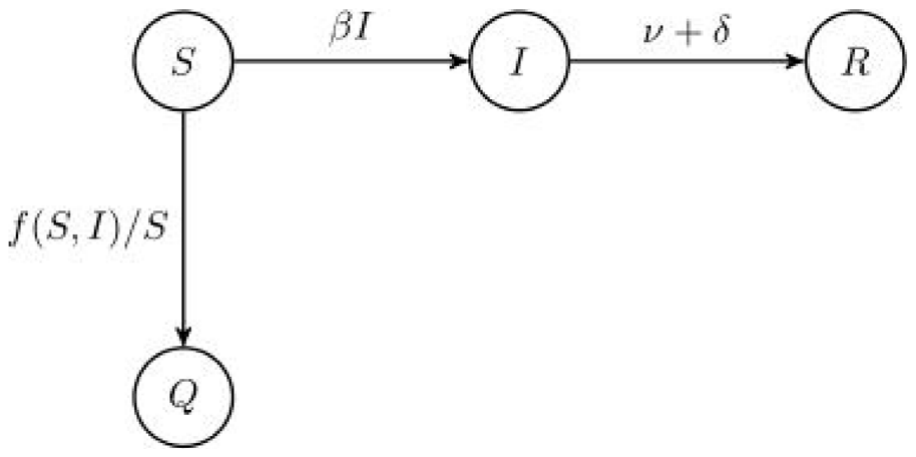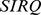 model schematic; short-lived outbreak.

We now describe the 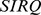 model for a short-lived outbreak of an emerging infectious disease. By short-lived we mean an outbreak on the scale of less than one year, where natural births and deaths have negligible effect on the disease dynamics. Susceptible individuals become infected at a rate proportional to the number of infected individuals. Infected individuals recover or die at a constant rate. Susceptible individuals choose to employ social distancing measures at a rate depending on the number of infected and susceptible individuals. The model is illustrated in Figure 6 and the model is a system of differential equations presented in Text S1.

The quantities 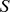, 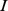, 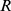, and 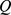 are the fraction of susceptible, infected, removed, and socially distanced individuals, respectively, in a population; 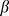 is the transmission rate; 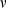 is the removal rate (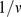 is the duration of infection); 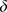 is the disease death rate; and 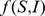 is the media influence function.

In this model, we assume that individuals who employ social distancing measures never return from their isolation (stop applying the social distancing measures). A priori, this assumption appears unrealistic. In the Results Section we illustrate (Figure 4) the corresponding short-lived model with return from social distancing. We show in our simulations that the severity of the outbreak (e.g. attack rate) is barely effected when allowing individuals to leave isolation for up to 4 hours each day. For this reason, the outcome of our model without the ability to stop social distancing will provide a good approximation to the more realistic case. In addition, we can determine formulas for key epidemiological characteristics for the strict social distancing model, which cannot be done with return from social distancing.

### Quantifying media influence

We incorporate three different types of media influence into our model. We derive explicit formulas for key epidemiological quantities across the entire range of parameter values. Again, determining these quantities formulaically, is a significant advantage of a low-dimensional mathematical model.

#### Media Influence Type 1

Susceptible individuals employ social distancing measures at a rate (heuristically probability) proportional to the number of reported infected individuals [14].

#### Media Influence Type 2

If the number of reported infections is small, then susceptible individuals employ social distancing measures at a rate proportional to the number of reported infections. As the number of reported infections increases, the rate saturates [10].

#### Media Influence Type 3

Susceptible individuals employ social distancing measures at a rate depending on both the number of susceptible and infected individuals. A susceptible looks at how many fellow citizens are susceptible. The fewer there are, the higher the rate that he or she will choose to employ social distancing measures. A mechanism of this type, where individuals follow the behavior of others, is postulated in [13].

The strength of each type of media influence is controlled by the parameter 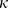, called the media influence intensity.

### Effectiveness of social distancing strategies

Although individuals believe they are immune from infection due to their social distancing actions, in reality they may not be. Masks are not 100% effective in preventing respiratory infections and aerosols can be transported through ducts in apartment buildings. In the model, these and other defects in perfect effectiveness are reflected by choosing a smaller media influence intensity 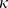.

### Delays in media reporting

We use our models to examine the effect of a government or media “holding-back” news of an outbreak. We examine the effects of two types of delays.

#### Delay Type 1

There is no reporting until some later time when the media starts reporting the number of infections starting at the beginning of the outbreak (historic data).

#### Delay Type 2

There is no reporting until some later time when the media starts reporting the current number of infections.

### Simulations

We illustrate our model with the different media influences by simulating an outbreak of a generic emerging infectious disease in a small city. We have in mind a virulent strain of avian influenza that is well adapted for human-to-human transmission. We select parameter values that mimic the outcome of the 1918 pandemic influenza [19]. In particular, the transmission rate 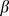 and disease related mortality, 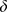, are selected to ensure 15% mortality for infected individuals and the basic reproduction number is 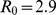 (see the Results Section). Individuals are assumed to be infectious for five days. We assume that the outbreak occurs in a small city with a population of 50,000 and that the disease is first reported on by health care workers and the media when there are 30 infected individuals. All of the parameters used in the model simulations are in Table 1.

Simulations for the non-strict social distancing were performed with varying 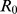 values, with 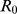 from 1.5 to 3.5. The disease related mortality and the length of the infectious period were those in Table 1, while the transmission rate was allowed to change.

### Mathematical analysis

Toward our goal of understanding the effects of social distancing, we derive analytic expressions for several key epidemiological characteristics of the models. We then analyze the dependence of these characteristics on the strength of the media influence intensity. These results are extremely general; the formulas and dependencies hold for all parameter values.

## Supporting Information

Figure S1

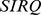
 **model schematic; long-lived outbreak.**
(TIF)

Figure S2
**Long-lived model; 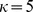; graphs of 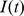 of different lengths of delays of Type 1.** Graphs of 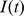 with media influence (a) 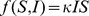 (b) 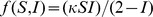 (c) 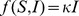. From the bottom up (at 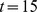): delay 0, 2, 7, 12 days. These are compared with the classical 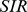 model with no media influence (“top” black curve).(TIF)

Table S1
**Excess morbidity caused by leaving social distancing for varying time outside of isolation and varying 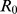 (by varying 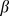); 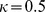.**
(PDF)

Table S2
**Excess morbidity caused by leaving social distancing for varying time outside of isolation and varying 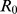 (by varying 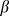); 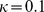.**
(PDF)

Text S1 **Supporting information.**
(PDF)
