# Supplementary figures and images for: Get the News Out Loudly and Quickly: The Influence of the Media on Limiting Emerging Infectious Disease Outbreaks
Source: PLoS One. 2013 Aug 26;8(8):e71692. doi: 10.1371/journal.pone.0071692 (PMC3753329; doi:10.1371/journal.pone.0071692)

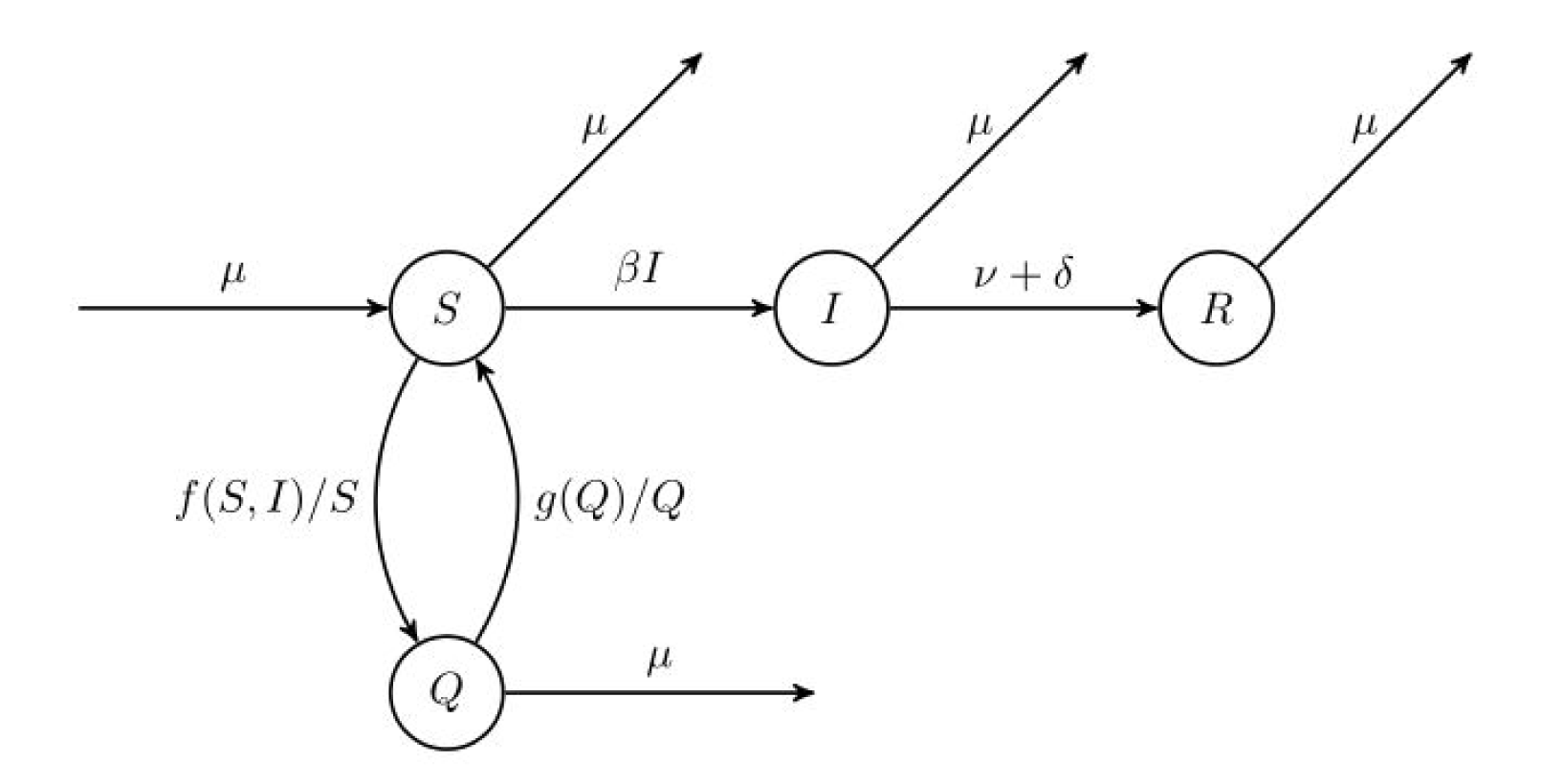

Supplement: Figure S1 — model schematic; long-lived outbreak. (TIF) [file pone.0071692.s001.tif]

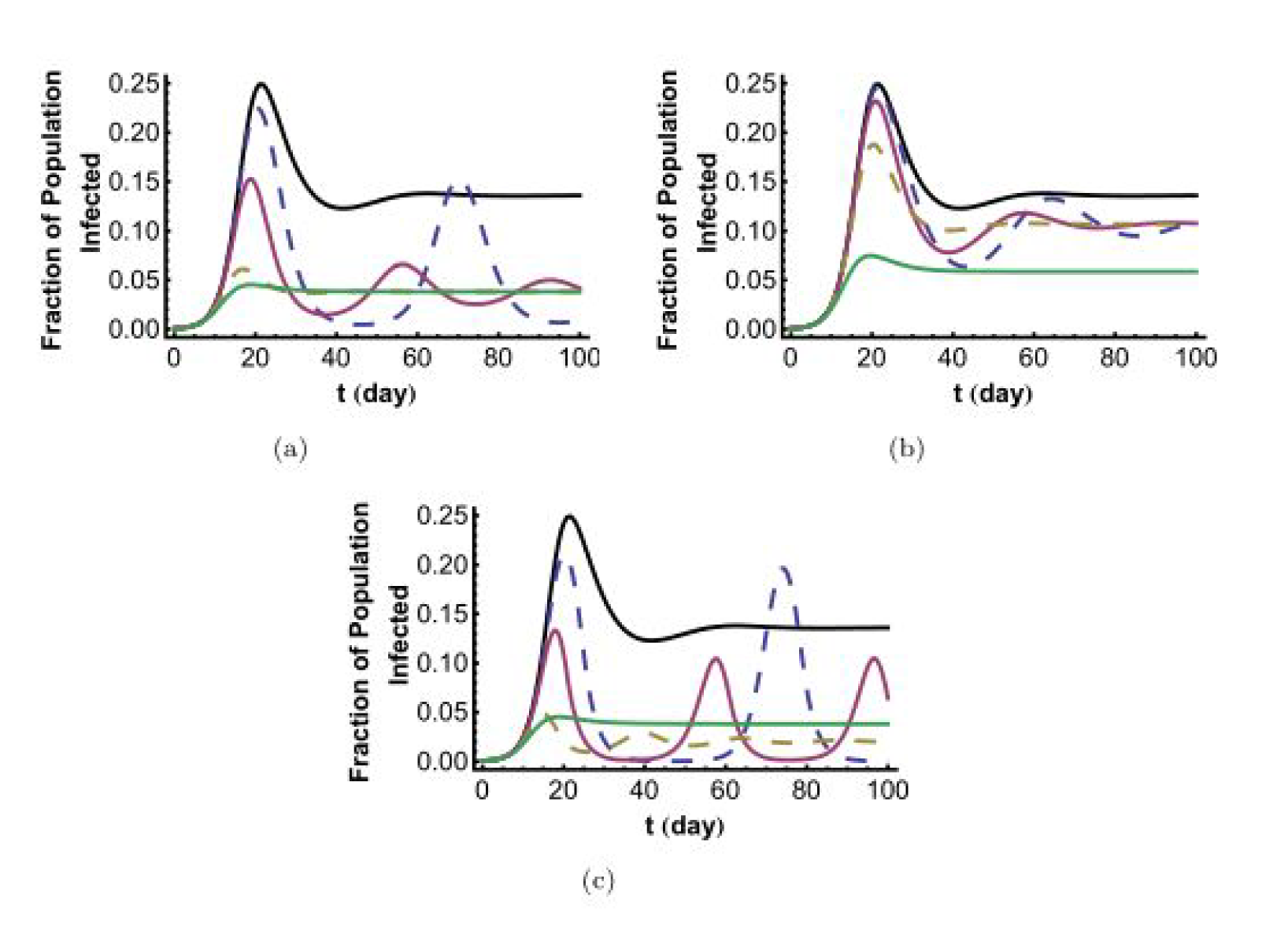

Supplement: Figure S2 — Long-lived model; ; graphs of of different lengths of delays of Type 1. Graphs of with media influence (a) (b) (c) . From the bottom up (at ): delay 0, 2, 7, 12 days. These are compared with the classical model with no media influence (“top” black curve). (TIF) [file pone.0071692.s002.tif]
