# Supplementary material for: Get the News Out Loudly and Quickly: The Influence of the Media on Limiting Emerging Infectious Disease Outbreaks
Source: PLoS One. 2013 Aug 26;8(8):e71692. doi: 10.1371/journal.pone.0071692 (PMC3753329; doi:10.1371/journal.pone.0071692)
Supplement: Table S1 — Excess morbidity caused by leaving social distancing for varying time outside of isolation and varying (by varying ); . (PDF) [file pone.0071692.s003.pdf]

Table S1. Excess morbidity caused by leaving social distancing for varying time outside of isolation and varying R_0 (by varying β); $\kappa = 0.5$.

R_0	Time Outside (hours)	Media Influence Function 1	Media Influence Function 2	Media Influence Function 3
1.5	2	1%	2%	1%
	4	3%	4%	2%
	8	7%	7%	6%
2	2	3%	2%	2%
	4	6%	5%	4%
	8	13%	10%	9%
2.5	2	4%	3%	3%
	4	8%	6%	7%
	8	16%	12%	16%
3	2	4%	4%	4%
	4	9%	8%	9%
	8	18%	14%	22%
3.5	2	5%	4%	6%
	4	10%	7%	12%
	8	20%	13%	27%
