# Supplementary material for: Get the News Out Loudly and Quickly: The Influence of the Media on Limiting Emerging Infectious Disease Outbreaks
Source: PLoS One. 2013 Aug 26;8(8):e71692. doi: 10.1371/journal.pone.0071692 (PMC3753329; doi:10.1371/journal.pone.0071692)
Supplement: Table S2 — Excess morbidity caused by leaving social distancing for varying time outside of isolation and varying (by varying ); . (PDF) [file pone.0071692.s004.pdf]

Table S2. Excess morbidity caused by leaving social distancing for varying time outside of isolation and varying R_0 (by varying β); $\kappa = 0.1$.

R_0	Time Outside (hours)	Media Influence Function 1	Media Influence Function 2	Media Influence Function 3
1.5	2	1%	1%	1%
	4	2%	2%	2%
	8	4%	3%	5%
2	2	2%	1%	2%
	4	3%	2%	4%
	8	6%	3%	7%
2.5	2	1%	1%	2%
	4	3%	2%	4%
	8	6%	4%	8%
3	2	2%	2%	2%
	4	3%	3%	5%
	8	6%	4%	10%
3.5	2	2%	1%	3%
	4	4%	2%	6%
	8	6%	3%	12%
