# Supplementary material for: Get the News Out Loudly and Quickly: The Influence of the Media on Limiting Emerging Infectious Disease Outbreaks
Source: PLoS One. 2013 Aug 26;8(8):e71692. doi: 10.1371/journal.pone.0071692 (PMC3753329; doi:10.1371/journal.pone.0071692)
Supplement: Text S1 — Supporting information. (PDF) [file pone.0071692.s005.pdf]

Supporting Information: Get the News Out Loudly and Quickly: The Influence of the Media on Limiting Emerging Infectious Disease Outbreaks

Anna Mummert[1,*], Howard (Howie) Weiss[2]

1 Mathematics Department, Marshall University, Huntington, WV, USA

2 Mathematics Department, Georgia Institute of Technology, Atlanta, Georgia, USA

$\ast$ **E-mail: mummerta@marshall.edu**

Mathematical Analysis: short-lived outbreak

Most proofs of the following statements are routine [6, 7] and sketches are provided only for the media influence type $f(S, I) = \kappa I$.

The general model for a short-lived outbreak is

$$\begin{aligned} \frac{dS}{dt} &= -\beta SI - f(S,I) + g(Q) \\ \frac{dI}{dt} &= \beta SI - \nu I - \delta I \\ \frac{dR}{dt} &= \nu I + \delta I \\ \frac{dQ}{dt} &= f(S,I) - g(Q) \end{aligned} \tag{S1}$$

subject to the initial conditions

$$S(0) = S_0 > 0, \quad I(0) = I_0 > 0, \quad R(0) = 0, \quad Q(0) = 0,$$

where the total population is a constant, N, so that $1 = S + I + R + Q$. The disease transmission rate is β, the rate of disease caused mortality is δ, and the recovery rate is ν. The rate at which susceptible individuals choose to employ social distancing measures is $f(S, I)$, a function of the number of susceptible and infected individuals, and the rate at which socially distanced individuals leave their isolation (stop social distancing measures) is $g(Q)$.

As pointed out in the Results Section and illustrated in Figure 4 (see also Tables S1 and S2) it is reasonable to assume strict social distancing, $g(Q) = 0$, for the mathematical analysis.

Media Influence Function 1: $f(S, I) = \kappa IS$

The following properties of the corresponding model hold.

Lemma 1. *Let $f(S, I) = \kappa IS$. The following properties hold for system (S1).*

- *The model has equilibrium points $(S^*, 0, R^*, Q^*)$ for all $0 \leq S^* \leq 1$, with $R^* \geq 0$ and $Q^* \geq 0$.*
  - *The equilibrium point $(1, 0, 0, 0)$ is weakly attracting when $R_0 < 1$ and it is weakly repelling when $R_0 > 1$.*
  - *The equilibrium points $(S^*, 0, R^*, Q^*)$, with $S^* \neq 1$, are weakly attracting when $R_0 > 1$ and are weakly repelling when $R_0 < 1$.*
- *S^* satisfies the equation*

$$S^* = S_0 + \frac{\kappa + \beta}{\beta} I_0 - \frac{\nu + \delta}{\beta} \log\left(\frac{S_0}{S^*}\right).$$

- *The maximum number of infected individuals is*

$$I_{max} = I_0 + \frac{\beta}{\kappa + \beta} S_0 - \frac{\nu + \delta}{\kappa + \beta} - \frac{\nu + \delta}{\kappa + \beta} \log\left(\frac{\beta S_0}{\nu + \delta}\right).$$

- *The total number of individuals who become infected is*

$$I_{total} = \left(\frac{\nu + \delta}{\kappa + \beta}\right) \log\left(\frac{S_0}{S^*}\right).$$

- *The number who chose to employ social distancing measures is*

$$(S_0 - S^*)\left(\frac{\kappa}{\kappa + \beta}\right).$$

- *I_{total} is decreasing functions of κ; when $R_0 > 1$, I_{max} is decreasing functions of κ; the total number who chose to employ social distancing measures is an increasing function of κ.*

Media Influence Type 2: $f(S, I) = \dfrac{\kappa IS}{2 - I}$

The following properties hold for the corresponding model.

Lemma 2. *Let $f(S,I) = (\kappa I S)/(2-I)$. The following properties hold for system (S1).*

- *The model has equilibrium points $(S^*, 0, R^*, Q^*)$ for all $0 \le S^* \le 1$.*
  - *The equilibrium point $(1,0,0,0)$ is weakly attracting when $R_0 < 1$ and it is weakly repelling when $R_0 > 1$.*
  - *The equilibrium points $(S^*, 0, R^*, Q^*)$, with $S^* \ne 1$, are weakly attracting when $R_0 > 1$ and are weakly repelling when $R_0 < 1$.*
- *S^* satisfies the equation*

$$S^* = S_0 + I_0 - \frac{\nu+\delta}{\beta} \log\left(\frac{S_0}{S^*}\right) - \frac{\kappa}{\beta} \log\left(\frac{2-I_0}{2}\right).$$

- *The maximum number of infected individuals is*

$$I_{max} = S_0 + I_0 - \frac{\nu+\delta}{\beta} \log\left(\frac{\beta S_0}{\nu+\delta}\right) - \frac{\kappa}{\beta} \log\left(\frac{2-I_0}{2-I_{max}}\right).$$

- *When $R_0 > 1$, I_{max} is decreasing function of κ.*

Media Influence Type 3: $f(S,I) = \kappa I$

We need to consider two scenarios: the fraction of susceptible individuals becomes zero in finite time or the fraction remains positive for all time. The second case occurs for sufficiently strong media influence κ, for κ larger than some threshold κ_*. The first case occurs for all smaller media influence values.

Lemma 3. *The threshold value κ_* is the unique positive solution to*

$$1 - \left(\frac{\kappa+\nu+\delta}{\beta}\right) \log\left(\frac{\kappa+\beta S_0}{\kappa}\right) = 0.$$

In particular, the value κ_* has the following properties:

1. *If $0 \le \kappa < \kappa_*$, then $S(t) > 0$ for all $t \ge 0$.*
2. *If $\kappa > \kappa_*$, then there exists a t_{crit}, with $0 < t_{crit} < \infty$, such that $S(t) > 0$ for all $0 < t < t_{crit}$, and $S(t_{crit}) = 0$.*

Proof. Divide dS/dt by dI/dt, separate variables, integrate, and use the initial conditions to solve for the integration constant to find that

$$I(t) + S(t) = 1 - \left(\frac{\kappa + \nu + \delta}{\beta}\right) \log\left(\frac{\kappa + \beta S_0}{\kappa + \beta S(t)}\right). \tag{S2}$$

Then κ_* is the unique positive κ whose long-term solution has both $I(\infty)$ and $S(\infty)$ equal to 0. When $\kappa < \kappa_*$, the long-term solution will have $I(\infty) = 0$ and $S(\infty) > 0$. When $\kappa > \kappa_*$ the long-term solution will have $I(\infty) = 0$ and $S(\infty) < 0$. □

The following properties hold for the corresponding model.

Lemma 4. *Let $f(S, I) = \kappa I$ with $0 \leq \kappa < \kappa_*$. The following properties hold for system (S1).*

- *The model has equilibrium points $(S^*, 0, R^*, Q^*)$ for all $0 \leq S^* \leq 1$, with $R^* \geq 0$ and $Q^* \geq 0$.*
- *S^* satisfies the equation*
$$S^* = 1 - \frac{\kappa + \nu + \delta}{\beta} \log\left(\frac{\kappa + \beta S_0}{\kappa + \beta S^*}\right).$$
- *When $R_0 > 1$, the maximum number of infected individuals is*
$$I_{max} = 1 - \frac{\nu + \delta}{\beta} - \frac{\kappa + \nu + \delta}{\beta} \log\left(\frac{\kappa + \beta S_0}{\kappa + \nu + \delta}\right).$$
- *The total number of individuals who become infected is*
$$I_{total} = \frac{\nu + \delta}{\beta} \log\left(\frac{\kappa + \beta S_0}{\kappa + \beta S^*}\right).$$
- *The number who chose to employ social distancing measures is*
$$\frac{\kappa}{\beta} \log\left(\frac{\kappa + \beta S_0}{\kappa + \beta S^*}\right).$$
- *I_{total} is decreasing functions of κ; when $R_0 > 1$, I_{max} is decreasing functions of κ; the total number who chose to employ social distancing measures is an increasing function of κ.*

Proof. The equilibrium points can be determined by setting each of the model equations in system (S1) equal to 0 and solving for S, I, R, and Q.

In equation (S2), use that the equilibrium point has $I^* = 0$ to find the equation for S^*.

Using Lemma 8, the maximum number of infected individuals occurs when $S = \dfrac{\nu + \delta}{\beta}$. Use this value of S in equation (S2), to determine I_{max}.

Divide dS/dt by dR/dt, separate variables, integrate, and use the initial conditions to solve for the integration constant to find the relationship between $S(t)$ and $R(t)$. Insert the long-term solution S^* into this equation to find I_{total}.

Divide dS/dt by dQ/dt, separate variables, integrate, and use the initial conditions to solve for the integration constant to find the relationship between $S(t)$ and $Q(t)$. Insert the long-term solution S^* into this equation to find the total number who employ social distancing measures.

The fact that maximum number of infected individuals at one time during an outbreak is a decreasing function of κ, the total number of infections during an outbreak, the attack rate, is a decreasing function of κ, and that the total number of individuals who choose to employ social distancing measures is an increasing function of κ can be shown by differentiation. For example, the derivative

$$\frac{dI_{total}}{d\kappa} = -\frac{(\nu + \delta)(S_0 - S^*)}{(\kappa + \beta S_0)(\kappa + \beta S^*)} < 0,$$

because, by the model equations, $S(t)$ is a decreasing function, meaning $S_0 > S^*$. □

Lemma 5. *Let $f(S, I) = \kappa I$ with $0 \leq \kappa < \kappa_*$. The following properties hold for system (S1).*

- *The equilibrium point $(1, 0, 0, 0)$ is weakly attracting when $R_0 < 1$ and it is weakly repelling when $R_0 > 1$.*
- *The equilibrium points $(S^*, 0, R^*, Q^*)$, with $S^* \neq 1$, are weakly attracting when $R_0 > 1$ and are weakly repelling when $R_0 < 1$.*

Proof. To determine local stability of the equilibrium points, compute the eigenvalues of the Jacobian matrix for system (S1). For the disease-free equilibrium three of the eigenvalues are 0, and the fourth is $\lambda = \beta - \nu - \delta$. Thus $\lambda > 1$ when $R_0 > 1$ and $\lambda < 1$ when $R_0 < 1$. For the equilibrium points $(S^*, 0, R^*, Q^*)$, again three eigenvalues are 0, while the fourth is $\lambda = \beta S^* - \nu - \delta$. □

For the case of $\kappa > \kappa_*$, at some time t_{crit} the fraction of susceptible individuals is 0. After this moment, the fraction remains 0, as no individuals are born or move into the susceptible class. Mathematically, we change the system of ordinary differential equations (S1) to:

$$\frac{dS}{dt} = \begin{cases} -\beta SI - \kappa I & 0 \le t \le t_{crit} \\ 0 & t > t_{crit} \end{cases} \tag{S3}$$
$$\frac{dI}{dt} = \beta SI - \nu I - \delta I$$
$$\frac{dQ}{dt} = \begin{cases} \kappa I & 0 \le t \le t_{crit} \\ 0 & t > t_{crit} \end{cases}$$
$$\frac{dR}{dt} = \nu I + \delta I \ .$$

Using the ideas similar to those in the proof of Lemma 4, the following properties hold.

Lemma 6. *Let $f(S, I) = \kappa I$ with $\kappa > \kappa_*$. The following properties hold for system (S1).*

- *The total number of individuals who become infected is*

$$I_{total} = 1 - \frac{\kappa}{\beta} \log\left(\frac{\kappa + \beta S_0}{\kappa}\right).$$

- *The number who chose to employ social distancing measures is*

$$\frac{\kappa}{\beta} \log\left(\frac{\kappa + \beta S_0}{\kappa}\right).$$

Proof. Substitute $S(t) = 0$ into equation (S2) to find the fraction of individuals in the infected class, I_{crit}, at t_{crit}. Similarly, use the relations between $S(t)$, $R(t)$, and $Q(t)$ to find the fraction of individuals in the R class, R_{crit}, and in the Q class, Q_{crit}, at t_{crit} . Then the total number of individuals who become infected is the sum $I_{crit} + R_{crit}$. □

Lemma 7. *Let $\kappa > \kappa_*$. System (S3) has two equilibrium points $(1, 0, 0, 0)$ and $(0, 0, R^*, Q^*)$.*

- *The equilibrium point $(1, 0, 0, 0)$ is weakly attracting when $R_0 < 1$, and it is weakly repelling when $R_0 > 1$.*
- *The equilibrium point $(0, 0, R^*, Q^*)$ is weakly attracting.*

Proof. The long-term equilibrium point in the case with $\kappa > \kappa_*$ corresponds to the equilibrium point of system (S3) after t_{crit}. Three of the eigenvalues of the corresponding Jacobian matrix are 0, while the fourth is $-(\nu + \delta)$. □

Delays in media reporting

We examine the effect of a government or media "holding-back" news of an outbreak, and first reporting the number of infections or deaths after the outbreak has progressed. We examine the effects of two types of delays on the media reporting functions.

Delay Type 1:

$$\begin{cases} 0 & t \leq \tau \\ f(S(t-\tau), I(t-\tau)) & t > \tau \end{cases}.$$

Delay Type 2:

$$\begin{cases} 0 & t \leq \tau \\ f(S(t), I(t)) & t > \tau \end{cases}.$$

Modeling a long-lived outbreak

The model equations are

$$\begin{aligned} \frac{dS}{dt} &= \mu(1-S) - \beta SI - f(S,I) + g(Q) \\ \frac{dI}{dt} &= \beta SI - \nu I - \delta I - \mu I \\ \frac{dR}{dt} &= \nu I + \delta I - \mu R \\ \frac{dQ}{dt} &= f(S,I) - g(Q) - \mu Q \end{aligned} \tag{S4}$$

subject to the initial conditions

$$S(0) = S_0 > 0, \quad I(0) = I_0 > 0, \quad R(0) = 0, \quad Q(0) = 0,$$

where the total population is a constant, N, so that $1 = S + I + R + Q$. The natural birth-death rate is μ, the disease transmission rate is β, the rate of disease caused mortality is δ, and the recovery rate

is ν. The rate at which susceptible individuals choose to employ social distancing measures is $f(S, I)$, a function of the number of susceptible and infected individuals, and the rate at which employ social distancing measures individuals leave isolation is $g(Q)$. The model schematic is given in Figure S1.

We assume that the function $f(S, I)$ is monotonically increasing in I and that $f(S, 0) = 0$. We also assume that the number of individuals reported by the media is proportional to the actual number of infected individuals, I.

We assume that individuals employ social distancing measures for a time of $1/\gamma$. Thus $g(Q) = \gamma Q$.

Lemma 8. *The model (S4) has the following properties.*

1. *The basic reproductive number is*
$$R_0 = \frac{\beta S_0}{\nu + \delta + \mu}.$$

2. *If $R_0 > 1$, the maximum daily disease prevalence, I_{max}, occurs when the number of susceptible individuals is*
$$S = \frac{\nu + \delta + \mu}{\beta}.$$

Proof. To determine the individual reproduction number R_0, verify that the equation dI/dt will be positive when $(\beta S_0)/(\nu + \delta + \mu)$ is greater than 1, and it will be negative when $(\beta S_0)/(\nu + \delta + \mu)$ is less than 1.

Similarly, if $R_0 > 1$, then verify that $dI/dt = 0$ when the number of infected individuals will achieve a maximum value, occurs when $(\beta S_0)/(\nu + \delta + \mu) = 1$. □

Also, if $R_0 > 1$, then the maximum number of infected individuals, I_{max}, occurs when the number of susceptible individuals is
$$S = \frac{\nu + \delta + \mu}{\beta}.$$

It is interesting to observe that the maximum number of infected individuals will happen when the number of susceptible individuals reaches a particular value, which does not depend on the media influence intensity. With a stronger media influence, more individuals choose to employ social distancing measures meaning that the value of S will be achieved earlier in the outbreak. Also, the maximum number of infected individuals will decrease.

In the examples provided below, we assume that thirty individuals are initially infected, for example

while traveling abroad, and return to a small city with total population 50,000. In this case, $S_0 \approx 1$, and the basic reproductive number could be written as

$$R_0 = \frac{\beta}{\nu + \delta + \mu}.$$

Media Influence Function 1: $f(S, I) = \kappa IS$

The following properties hold for the corresponding model.

Lemma 9. *Let $f(S, I) = \kappa IS$. The following properties hold for system (S4).*

- *The model has the disease-free equilibrium point* $(1, 0, 0, 0)$.
- *The model has the endemic equilibrium point (S^*, I^*, R^*, Q^*), where, setting $a_2 = \kappa\mu + \beta(\mu + \gamma)$,*

$$\begin{aligned} S^* &= \frac{\nu + \mu + \delta}{\beta} \\ I^* &= \frac{\mu(\mu + \gamma)(1 - S^*)}{a_2 S^*} \\ R^* &= \frac{(\mu + \gamma)(\nu + \delta)(1 - S^*)}{a_2 S^*} \\ Q^* &= \frac{\kappa\mu(1 - S^*)}{a_2} \quad . \end{aligned}$$

- *The equilibrium point* $(1, 0, 0, 0)$ *is weakly attracting when $R_0 < 1$, and it is a saddle point when $R_0 > 1$.*

Numerical simulations indicate that the endemic equilibrium point is globally stable.

Media Influence Function 2: $f(S, I) = \dfrac{\kappa IS}{2 - I}$

The following properties hold for the corresponding model.

Lemma 10. *Let $f(S, I) = (\kappa IS)/(2 - I)$. The following properties hold for system (S4).*

- *The model has the disease-free equilibrium point* $(1, 0, 0, 0)$.
- *The model has two additional non-negative equilibrium points (S^*, I^*, R^*, Q^*).*
- *The equilibrium point* $(1, 0, 0, 0)$ *is weakly attracting when $R_0 < 1$, and it is a saddle point when $R_0 > 1$.*

Media Influence Function 3: $f(S, I) = \kappa I$

As in the case of the short-lived model, there is a threshold $\kappa_* > 0$ such that if $0 \leq \kappa < \kappa_*$, then $S(t) > 0$ for all $t \geq 0$, and if $\kappa > \kappa_*$, then there exists a t_{crit}, with $0 < t_{crit} < \infty$, such that $S(t) > 0$ for $0 < t < t_{crit}$, and $S(t_{crit}) = 0$. At some time all individuals have chosen to employ social distancing measures or have become infected, which would be expected for a frightening virulent disease. Due to the complicated form of model (S4), it is not possible to determine an explicit formula for κ_*; it is possible to determine it numerically[1]. For $\kappa > \kappa_*$, we mathematically rewrite system (S4), as was done for the short-lived model.

Unlike the short-lived outbreak, the number of susceptible individuals does not remain at 0. Eventually, a sufficient number of infected individuals would recover so that $\mu + g(Q) > \kappa I$, causing $dS/dt > 0$, meaning the fraction of susceptible individuals would increase.

The following properties hold for the corresponding model.

Lemma 11. *Let $f(S, I) = \kappa I$, with $0 \leq \kappa < \kappa_*$. The following properties hold for system (S4).*

- *The model has the disease-free equilibrium point $(1, 0, 0, 0)$.*
- *The model has the endemic equilibrium point (S^*, I^*, R^*, Q^*), where, setting $a_1 = \delta(\mu + \gamma) + \gamma(\nu + \mu) + \mu(\kappa + \nu + \mu)$,*

$$\begin{aligned} S^* &= \frac{\nu + \delta + \mu}{\beta} \\ I^* &= \frac{\mu(\mu + \gamma)(1 - S^*)}{a_1} \\ R^* &= \frac{(\mu + \gamma)(\nu + \delta)(1 - S^*)}{a_1} \\ Q^* &= \frac{\kappa\mu(1 - S^*)}{a_1} \ . \end{aligned}$$

- *The equilibrium point $(1, 0, 0, 0)$ is weakly attracting when $R_0 < 1$, and it is a saddle point when $R_0 > 1$.*

Numerical simulations indicate that the endemic equilibrium point is globally attracting for $\kappa < \kappa_*$.

For $\kappa > \kappa_*$, the simulations indicate that the system also eventually tends to some positive endemic equilibrium point (S^*, I^*, R^*, Q^*).

[1] For the model simulation considered here with $\mu = 0.000035$ and $\gamma = 1/7$, $\kappa_* = 0.1123$.

Delays in media reporting

Warning! Reporting historic data can have the opposite effect than intended. Figure S2 shows the dynamics of a long-lived outbreak with media reporting Delay Type 1 for a disease with natural birth-death rate $\mu = 0.08$ and $\gamma = 1/7$. It illustrates the striking property that a delay of 12 days can result in a resurgence in the prevalence (see especially media influence type 3). Thus public health officials and the media need to be cognizant that they are not causing additional morbidity and mortality by releasing historic data.

Non-strict social distancing

The excess morbidity caused by leaving social distancing for varying time outside of isolation and varying R_0 can be seen in Tables S1 and S2 for $\kappa = 0.5$ and $\kappa = 0.1$, respectively.

Figure Legends

Figure S1. $SIRQ$ model schematic; long-lived outbreak.

Figure S2. Long-lived model; $\kappa = 5$; graphs of $I(t)$ of different lengths of delays of Type 1. Graphs of $I(t)$ with media influence (a) $f(S,I) = \kappa IS$ (b) $f(S,I) = (\kappa SI)/(2-I)$ (c) $f(S,I) = \kappa I$. From the bottom up (at $t = 15$): delay 0, 2, 7, 12 days. These are compared with the classical SIR model with no media influence ("top" black curve).

Table Legends

Table S1. Excess morbidity caused by leaving social distancing for varying time outside of isolation and varying R_0 (by varying β); $\kappa = 0.5$.

Table S2. Excess morbidity caused by leaving social distancing for varying time outside of isolation and varying R_0 (by varying β); $\kappa = 0.1$.
